# Metabolic Responses and Resilience to Environmental Challenges in the Sedentary Batrachoid *Halobatrachus didactylus* (Bloch & Schneider, 1801)

**DOI:** 10.3390/ani13040632

**Published:** 2023-02-11

**Authors:** Juan Manuel Molina, Andreas Kunzmann, João Pena Reis, Pedro Miguel Guerreiro

**Affiliations:** 1 Instituto Argentino de Oceanografía (CONICET), Bahía Blanca B8000, Argentina; 2 Leibniz-Zentrum für Marine Tropenforschung (ZMT), 28359 Bremen, Germany; 3 Centro de Ciências do Mar (CCMAR), Universidade do Algarve, 8005-139 Faro, Portugal

**Keywords:** fish physiology, climate change, hypoxia tolerance, temperature tolerance, metabolic rate, aerobic scope

## Abstract

**Simple Summary:**

In the context of climate change, warming of the seas and expansion of hypoxic zones are challenges that most species of fish are, or will be subjected to. We provide first-time estimates on the metabolic rates, thermal, and oxygen-related limits for *Halobatrachus didactylus*, a coastal sedentary fish that lives in intertidal environments of the Northeast Atlantic. The metrics obtained in this study prove that *H. didactylus* is remarkably resilient to acute environmental variations in temperature and oxygen content, which might enable it to adapt to the extreme abiotic conditions forecasted for the world’s oceans in the near future.

**Abstract:**

In the context of climate change, warming of the seas and expansion of hypoxic zones are challenges that most species of fish are, or will be subjected to. Understanding how different species cope with these changes in their environment at the individual level can shed light on how populations and ecosystems will be affected. We provide first-time estimates on the metabolic rates, thermal, and oxygen-related limits for *Halobatrachus didactylus*, a coastal sedentary fish that lives in intertidal environments of the Northeast Atlantic. Using respirometry in different experimental designs, we found that this species is highly resistant to acute thermal stress (CT_max_: 34.82 ± 0.66 °C) and acute hypoxia (P_crit_: 0.59–1.97 mg O_2_ L^−1^). We found size-specific differences in this stress response, with smaller individuals being more sensitive. We also quantified its aerobic scope and daily activity patterns, finding this fish to be extremely sedentary, with one of the lowest standard metabolic rates found in temperate fish (SMR: 14.96 mg O_2_ kg^−1^h^−1^). *H. didactylus* activity increases at night, when its metabolic rate increases drastically (RMR: 36.01 mg O_2_ kg^−1^h^−1^). The maximum metabolic rate of *H. didactylus* was estimated to be 67.31 mg O_2_ kg^−1^h^−1^, producing an aerobic scope of 52.35 mg O_2_ kg^−1^h^−1^ (77.8% increase). The metrics obtained in this study prove that *H. didactylus* is remarkably resilient to acute environmental variations in temperature and oxygen content, which might enable it to adapt to the extreme abiotic conditions forecasted for the world’s oceans in the near future.

## 1. Introduction

Climate warming produces changes in water temperature and, consequently, in oxygen availability, which forces marine ectotherms to acclimate and, in the long-term, adapt by means of metabolic and behavioral changes [1,2,3]. According to the most favorable prediction of the Special Report on Emissions Scenarios, by the year 2100, the oceans’ temperature and oxygen minimum zones will increase [4]. These changes in the abiotic conditions of the oceans are expected to produce severe impacts on marine organisms [4,5]. Most of these changes will occur in coastal areas, often in systems already affected by important natural fluctuations in temperature and oxygen levels [1,3,4,5]. One example of such environments are coastal lagoons, which have a major ecological role, functioning as nurseries for pelagic species and habitats for many sedentary fish that also provide important ecosystem services [6,7].

In this context, increasing our understanding of thermal and hypoxia tolerance and the stress responses of fish on an organismal level is of high relevance. This is also important from an economic point of view, for fisheries and aquaculture [5]. Despite the relevance of this type of research, information on the thermal and oxygen-related limits is scarce or lacking for a large proportion of fish species [8]. Temperature and hypoxia tolerance have been traditionally approached by measuring the critical thermal maximum (CT_max_) and critical oxygen partial pressure (P_crit_) [9]. CT_max_ is a measure of an animal’s upper thermal tolerance limit. Its determination consists of exposing groups of fish to a constant increase in temperature until some form of physiological failure is evident (e.g., loss of equilibrium, gill ventilation stops, inability to move or react) [8,10]. The biological threshold meaning of this metric is “the thermal point at which locomotory activity becomes disorganized and the animal loses its ability to escape from conditions that will promptly lead to its death” [10] (p. 1562). CT_max_ is considered to be ecologically relevant, with relatively high repeatability, and is methodologically straight-forward, making it relevant for continued use as a metric for acute thermal tolerance [8,9,11]. Aside from CT_max_, which has been employed extensively and provides an important metric of thermal tolerance, other methods have also been employed to measure the effect of increased temperature on the physiology of fish [12]. Temperature ramps, for example, have been employed to measure the effect of acutely increasing the temperature on fish, and are considered to be good at replicating natural stressors like those affecting migrant fish or coastal and intertidal pool inhabitants [13,14,15,16,17].

P_crit_ represents the oxygen partial pressure at which oxygen consumption switches from a regulator to conformer on oxygen partial pressure [18,19,20]. P_crit_ has also been given the biological threshold meaning of a value below which activity can no longer be sustained aerobically [20,21,22]. Several authors have challenged this view of P_crit_ as the sole metric to look into hypoxia, raising concerns about inconsistent or incorrectly applied methodologies as well as suggesting alternative metrics [23,24]. Methodologically, however, alternatives to P_crit_ have not been fully explored, and the theoretical alternatives are not widespread enough to be validated by their use on different species [24]. Regardless of these discrepancies, the basic methodology is subjecting fish to an ever-decreasing oxygen tension while measuring their oxygen consumption. The tension at which the fish consumption of oxygen becomes dependent on the oxygen tension is considered the P_crit_ [20].

The European toadfish *Halobatrachus didactylus* is a sedentary species, with mostly sit-and-wait predatory habits, and is a member of the order Batrachoidiformes. This fish is distributed all along the Western Mediterranean coast [25] and the Central and Northeast Atlantic [26,27]. It has low commercial value, but is commonly caught by artisanal fishermen of southern Portugal and is sold at local markets [28]. *H. didactylus* can reach lengths of 50 cm in total, being one of the largest Batrachoididae species [29]. It inhabits shallow waters near coasts, estuaries, and coastal lagoons [30]. It prefers muddy and sandy sediments where it can burrow, and remains covered by sediment, algae, or rocks for long periods of time, waiting for prey or resting [31]. The species has been used as an experimental model for studies on toxicology, cardiac function, behavior, and spatial ecology [32,33,34,35]. Physiological and life-history traits influence how species respond to abiotic factors, which means that lifestyle alone could be an important proxy for how a certain species will respond to environmental change [12,36,37,38,39]. The benthic-associated life history traits of *H. didactylus* make it an inevitable subject of the particular stressors of its habitat, and its coastal distribution and association with intertidal mudflats subjects them to diurnal changes in temperature as a result of the tidal regime. These changes in temperature are of quite notable magnitudes (10–30 °C) [40]. *H. didactylus*, other batrachoids, and even other fish inhabiting these highly variable ecosystems, with a wide range of temperatures and oxygen levels, have developed physiological adaptations that allow them to be able to survive and reproduce. Despite the relative wealth of knowledge on *H. didactylus*, many of its physiological traits remain unknown or understudied, which are needed to fully understand their physiological resilience. The work presented here is the first of a series of studies in which we explore the effects of man-made climate change stressors on the physiology of *H. didactylus*.

In this paper, we aimed to quantify and determine the aerobic energy budget, thermal limits (CT_max_), and critical oxygen tension (P_crit_) of the Lusitanian toadfish *H. didactylus*.

## 2. Materials and Methods

### 2.1. Sourcing and Housing

Individuals of *H. didactylus* were sourced from the coastal lagoon Ria Formosa, located near Faro, in the Algarve region of Portugal, during December 2019 and January 2020. This lagoon is highly productive and suffers from considerable anthropogenic pressure and economic exploitation, but as a Natural Park, it has benefited from several protection measures against highly destructive commercial fishing methodologies [41]. *H. didactylus* individuals were captured using a beam trawl, at night, close to the shore or close to seagrass beds [42] at depths ranging from 1 to 2 m and for up to 15 min. The net was pulled by hand and when the catch was onboard, *H. didactylus* were immediately sorted by size and retained in containers with aerated seawater. Fish were then quickly transported to a common open circulation system fed with water pumped from the lagoon, located in the nearby Ramalhete Marine Station (University of Algarve), and allocated by size range in large acclimation tanks (volume 600 L, bottom area 1.3 m2). The fish were allowed to recover in these acclimation tanks for 1 month from the stress originated from the capture and transport. During this period, they were fed squid and mussels (every other day, 3% *w*/*w* tank biomass), and kept at natural temperature, salinity, and photoperiod (Jan 2020: 12.9 ± 0.28 °C; 35.1 ± 0.05 PSU; 10 light hours (L) and 14 no light hours (D); Feb 2020: 15.9 ± 0.11 °C; 35.6 ± 0.06 PSU; 11L:13D). As the species inhabits crevices and small caves, PVC tubes for shelter were provided, which were readily accepted. Only juvenile fish were employed in this study because of the confounding effects that gonadal development may produce on energy expenditure.

Each of the trials described in the following sections utilized independent samples, that is, once a fish was used for an experiment, it was returned to a different acclimation tank, and not used again for any other procedure. This protocol ensured independence of the results, albeit limiting the size range and number of individuals for each trial.

Fish were collected under special license from the Portuguese Agency for Nature Conservancy (ICNF; 7048/2020/DCBN/DAN) and the General Directorate for Marine Resources (DGRM; 1009/2019/DRI). Experiments were conducted following the guidelines established by the EU Directive 2010/63/EU and the Portuguese Decree Law No. 113/2013 on “The protection of animals used for scientific purposes”. The experimental design was previously approved by the CCMAR Ethical Committee for Managing Animal Welfare (ORBEA) and by the Portuguese Veterinary Authority (DGAV) under permit 009272. Fish manipulation was performed by accredited scientists in laboratory animal science by the Portuguese DGAV, following the FELASA category C recommendations.

### 2.2. Metabolic Rates Measurement Procedure

To estimate the metabolic rates (MRs) of *H. didactylus*, we measured the oxygen consumption by means of a respirometry array. In designing our respirometry system, we followed the guidelines outlined in [43]. This array consisted of four flow-through transparent respiration chambers made of acrylic (1.7 L and 2.5 L chambers, approximately between 20 and 55 respirometer:fish volume ratio depending on the fish size) that were outfitted with circulation and in/out flow control pumps, and placed inside 200 L tanks (measuring tanks). The tanks had top PVC covers to reduce the visual disturbance of the fish. The dissolved oxygen concentrations were measured inside each chamber with an optical oxygen sensor probe (Pyroscience GmbH, Germany) that directly fed data in 20 s intervals (computing an average of 20x 1 s data points) to a computer using the FirestingGo interface and dedicated Pyroscience software. Oxygen was measured in “respiration cycles” that consisted of two phases. In the first phase, the chamber was sealed, allowing the fish to consume the oxygen in the chamber (from 100% to a minimum of 85% O_2_ saturation). The second phase consisted of flushing the chamber with fresh sea water by means of an electrically activated pump. The system was configured to measure for 8 min and flush for 2 min. Animals measured for oxygen consumption were left unfed 48 h prior to the measurement to ensure a post absorptive state [9]. The number of cycles conducted (and therefore the total measuring time) varied depending on the experimental set-up performed, as described in the following sections. Similar set-ups are described in greater detail in [9,44,45].

### 2.3. Standard, Routine and Maximum Metabolic Rate

In this study, we estimated the standard, routine, and maximum metabolic rates (SMR, RMR, and MMR, respectively) of *H. didactylus*. Sixteen individuals (size range: 13.9–147.9 g) were selected to run the SMR and RMR determinations, while a different set of 12 animals (size range: 60.0–119.3 g) were selected for the MMR trial. For this work, and following the framework for respirometry research [9], SMR is considered to be the MR of a post-absorptive fish in a quiescent and calm state. The RMR is the consumption of oxygen of a fish that is calm, but performs at-will movements or other minor activities during the measurement [9]. For SMR and RMR, fish were simply transferred to the measuring tanks and into the respiration chambers at 9 am, and the oxygen measurement was started right away. These procedures lasted for 75 hs. For MMR determination, we followed the standard protocols outlined in the literature [9,43,46,47]. Briefly, fish were transferred to an annular pool (0.75 m in circumference) with water fed from the acclimation tanks, and chased around by gently touching the caudal region. The fish would swim in bursts for a short period and then remain motionless, a point at which further stimulus was provided. The fish were considered exhausted when they stopped reacting to tail pinching. Time until exhaustion (in minutes) and the number of laps completed were recorded. Bony fish have been shown to recuperate from exercise quickly, and such recovery can occur even when the fish is unresponsive, and when being moved from the exercise pool and inserted in the respirometry chamber [46,47,48,49]. For this reason, fish in this state were transferred to the measuring tanks and into the respiration chambers, and the oxygen measurement was started right away (less than 20 s). Respiration cycles in this case were inverted, with 2 min of measuring time and 8 min of flushing to allow the fish to recuperate properly, preventing fast depletion of oxygen within the sealed chamber [49]. This procedure lasted for 24 h.

We performed 24 h measurements (based on suggestions from the literature, e.g., [9,43,46]) to determine SMR, RMR, MMR and the time until the MR values stabilized after the initial handling and after exhausting exercise. These experiments were run at 15 °C with fish previously acclimated in the acclimation tanks (12–16 °C, see sourcing and housing section for more details on acclimation conditions).

After completing the respirometry trial, fish were measured for TL (mm), weight (g), and volume (L) before being returned to the acclimation tanks. The volume of each animal was measured using the water displacement technique, which consists of measuring the volume of water displaced by submerging the animal in a graded container.

### 2.4. Mass-Scaling Exponent

The dissolved oxygen (DO) data to calculate the mass-scaling exponent (ME) were taken from the measurements collected from the 16 individuals used in the SMR/RMR section. Data points were those corresponding to the stabilized and resting individuals. There are no external sexual dimorphisms in the juveniles of this species, and since the individuals were not euthanized, it was impossible to determine an exponent for females and males separately. All animals were at a length below sexual maturity, therefore, a separate exponent for adults was not calculated.

### 2.5. Critical Thermal Maximum (CT_max_) Trial

CT_max_ was estimated by subjecting 16 individuals (26–82 g) to a rapid increase in temperature until they lost equilibrium by recording the time and temperature. Fish were placed in an aquarium connected to a tank, outfitted with a circulation pump and a heating element, and left undisturbed for 6 h (double of what was determined in the SMR/RMR trial) before the trial started. The water temperature started at 15 °C (the acclimation temperature) and was increased by the heating element in the tank to provide a gradual and smooth increase in the water temperature in the aquarium, at a rate of approximately 1 °C each 20 min [12,15]. Oxygen saturation was monitored and maintained at 85–100% throughout the trial [9] with compressed air delivered through several aeration stones. Water temperature in the aquarium was increased until all fish had lost equilibrium, which was the CT_max_ endpoint [9]. When a fish lost equilibrium, the time and temperature were noted, and it was removed from the container, weighed, and measured (TL (mm), weight (g), and volume (L)). After these procedures, they were placed in a container with the same water. When the temperature naturally dropped to 15 °C, the fish were returned to the acclimation tanks. All except one fish recuperated from this trial.

### 2.6. Temperature Ramp

To further explore the effects of acute temperature stress as a result of tidal regime, we employed a temperature ramp protocol. This protocol was suggested by Vinagre et al. [15], and further employed in Campos et al. and Rangel and Johnson [12,50]. Thirty-two fish (size range: 13.2–159.1 g) were taken, eight at a time, from the acclimation tanks, and transferred to the respiration chambers of the measuring tanks of the respirometry array at 16:00 in the afternoon. The water temperature of the measuring tanks was 12 °C. According to the results of the aerobic scope section, fish were left in the chambers undisturbed for 6 h (double of what was determined in the SMR/RMR trial), with the flushing pump always on, so that their MR values stabilized. After this time, the computer-controlled water temperature was reduced to 8 °C over 4 h in order to further reduce the oxygen consumption. After 4 h at 8 °C, the temperature was increased. The increases were programmed so that the temperature would rise 2 °C in an hour, remain stable for another hour, and then rise again in the same stepwise manner up to 32 °C in the course of 24 h, while measuring the oxygen consumption. Temperature was left to descend to 28 °C before removing the fish from the chambers, measuring their TL (mm), volume (L), and weight (g). After these procedures, they were placed in a container with the same water. When the temperature naturally dropped to 15 °C, the fish were returned to the acclimation tanks.

### 2.7. Critical Oxygen Tension Trial

Acute hypoxia tolerance was estimated using eight individuals (43.7–81 g), which were taken from the acclimation tanks and transferred to the respiration chambers of the respirometry array at 07:00 in the morning, and left undisturbed for 6 h (double of what was determined in the SMR/RMR trial). The respirometry array was started with 100% O_2_ saturated water (8.5 mg O_2_ L^−1^ at 15 °C), and thereafter, the saturation was lowered 10% every 30 min. We achieved this by bubbling N_2_ in the measuring tank, which was covered with plastic bubble wrap to minimize the loss of N_2_, until the desired saturation point was reached. Oxygen saturation in the measuring tank was monitored using a handheld ProSolo Optical Dissolved Oxygen and Conductivity Meter fitted with an optical, non-consumptive DO sensor (YSI, Yellow Springs, OH, USA, https://www.ysi.com/prosolo-odoct (accessed on 15 January 2020)). Saturation was lowered until 2% saturation (0.25 mg O_2_ L^−1^ at 15 °C) was reached, or the fish lost equilibrium [9,20,24]. Loss of equilibrium in this species was readily apparent, as the fish tilted to one side on the gentle water flow of the chamber. Immediately after the fish lost equilibrium, or by the end of the trial (at 2% DO), the fish were removed from the chambers, weighed, and measured for TL and volume, and then returned to their acclimation tanks.

### 2.8. Data Analysis

The normality of the error structure of the oxygen data was assessed with a quantile–comparison plot, and the kurtosis and skewness tests as well as mean–variance plots. Certain distributions were normal (i.e., SMR, RMR, MMR, CT_max_ data) and others were log-transformed (i.e., ME, P_crit_, temperature ramp), after which the data fitted well to a normal distribution; therefore, we employed linear statistics for further analysis [51].

#### 2.8.1. Metabolic Rates

The dissolved oxygen level in the respiration chambers was plotted against time for each respirometry cycle of an individual fish and a regression slope was obtained. These regression slopes were used to calculate the metabolic rates, modified from Clark et al. [52] as:

MR = VO_2_ × VR × (M^ME^)**^−^**^1^ × t^−1^,

where MR is the metabolic rate of the fish, measured as mg O_2_ kg^−1^h^−1^; VO_2_ is the slope of the linear model fitted to the oxygen concentration (mg L^−1^) data from the start through to the end of each cycle; VR is the volume of the respirometer chamber (L) calculated as the total volume of the chamber minus the volume of each animal; M is the total wet body mass of the animal (kg); and t is the duration (hours) of the cycle. ME is the mass-scaling exponent. This exponent was estimated fitting a linear regression model using the logarithm of DO and fish weight (*n* = 28) and the slope (the ME exponent) was extracted from the model. This exponent was used to calculate MR in the SMR set of individuals to showcase the effectiveness of the calculated ME. An ANOVA, followed by a Tukey pairwise comparison, was used to test the possible differences in the MR between fish sizes [51].

To estimate the acclimation-to-respirometer times, we performed an ANCOVA test to detect the differences in the slopes between the cycles for each individual fish from the SMR/RMR trial during the 24 h of measurements [53]. The initial slopes were more pronounced, and gradually became smaller with the passage of time. Once the analysis found no significant differences between the slopes, the animal was assumed to be stable and resting. Time to acclimation was computed and averaged between all the fish that took part in the trial. This acclimation-to-respirometer time was estimated to be 3.2 h. This exact same analytical process was employed to determine how much time it took for the fish in the MMR experiment to recuperate from exhaustion and regain steady oxygen consumption.

In order to estimate the daily oscillations in the activity of this species, the MR data from the SMR/RMR trial were used to fit a sine wave function using non-linear regression (function nls of R base library). We used three error terms for harmonic oscillation in order to mechanistically characterize the daily fluctuations in activity, according to the formula:

Metabolic rate (mg O_2_ kg^−1^h^−1^) = amplitude × sine (2 × π × frequency × time of day + phase) + offset

Additionally, data were grouped obtaining a median, standard deviation, and the first and third quartiles per hour. SMR and RMR were determined following the recommendations of Remeyer and Rees, and Killen et al. [20,39] for standardized metabolic measurements and calculations. Briefly, for SMR, we opted for the median of the quantiles that placed SMR below the lowest 20% of the daylight (inactive phase) observations (q 0.2) for each fish. For RMR, the median of the quantiles that placed RMR above the highest 80% of the night (active phase) observations (q 0.8) for each fish were used [20].

To determine MMR, we employed the rolling regression method with a window width of 1 min [49]. Next, we used an ANCOVA test to detect the differences in the slopes between the cycles for each individual fish [53]. Oxygen consumption recorded for the highest significantly different period of measurements in the MMR trial was recorded per fish, and expressed as the median of the quantiles that placed MMR above the highest 80% of the measurements of all fish (q 0.8). We then plotted the MR vs. time using boxplots, with the significantly different time interval where MMR was found, followed by hourly intervals to visualize recovery. Finally, individual splines were fitted to each fish data points [51].

Aerobic scope and routine aerobic scope were calculated both as the differences and as the rate between MMR and SMR, and RMR and SMR, respectively [9,46,49,54].

The effect of size (as weight) in the MRs determined in this section was tested using linear regression [51].

Additionally, for MMR, the effect on the estimation of MR from the trainer and time being chased was also tested for significance. Stepwise deletion of non-significant terms in a linear model including all terms was the method employed [51]. Concrete examples and a more thorough description of this method are given elsewhere (e.g., [55,56]).

#### 2.8.2. CT_max_ and Acute Thermal Tolerance

A linear regression model was used to test whether CT_max_ changed with weight, given the normal distribution of the data [51].

The acute temperature exposure data were analyzed using recursive partitioning and regression trees (RPRT) [57]. This method is used to split the predictor variable/s (in our case, temperature and fish size) into statistically significant ranges within which the response variable (in our case, the MR) remains significantly constant. Briefly, the algorithm starts with the complete dataset and tests if it should be split. If the null hypothesis is rejected, the variable is split into two new ranges. The steps will be repeated within both of the new ranges (hence the “recursive partitioning”). The tree stops growing, if in all partitions a global null hypothesis of independence cannot be rejected [57]. The procedure defines “nodes” or divergent values in the continuous variable from which the statistically different ranges are grouped [57], making it possible to discern where the changes in MR occur in the ranges employed. Using the R package rpart, with the “anova” method, we ran a RPRT using MR as the response variable with fish weight and temperature as the predictor variables to partition them into ranges [57]. The MR of each size class at each range of temperature was compared by ANOVA and Tukey pairwise comparisons [51].

#### 2.8.3. P_crit_

P_crit_ for each fish was calculated following the methods proposed by [20,24] (herein presented as the “α method” and the “LLO method”, respectively). The former study suggested the estimation of α as a species and temperature specific constant based on the metabolic rate dependence of P_crit_ (P_crit_ = MR/α). Oxygen consumption was monitored as the O_2_ partial pressure declined and was divided by the corresponding O_2_ partial pressure to obtain α0. The mean of the three highest values of α0 was designated as α. The later study defined P_crit_ as the O_2_ partial pressure at which O_2_ consumption dropped below its SMR. A linear relationship between O_2_ consumption and O_2_ partial pressure was fitted (to data collected after O_2_ consumption fell below that individual’s SMR) and from this relationship, the O_2_ partial pressure where O_2_ consumption equals SMR is considered as P_crit_. We performed both calculations for each of the eight fish in our critical oxygen tension trial, and then averaged the results to obtain an estimate for the species’ P_crit_.

All tests used a significance level of α ≤ 0.05. Data were processed using R statistical software [58] using scripts specifically developed for this purpose and functions in the respirometry, FSA, and lme4 packages.

## 3. Results

### 3.1. Mass-Scaling Exponent of Oxygen Consumption

**Figure 1:**
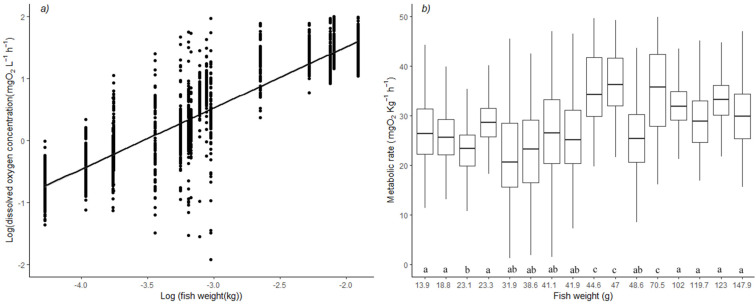
(**a**) Scatterplot of the log dissolved oxygen concentration vs. log weight of *Halobatrachus didactylus*. The line represents a linear model fit to the data. (**b**) Mass-corrected routine metabolic rate for *H. didactylus* individuals of varying weights. Different letters below the boxplot indicate statistical results from ANOVA (F-statistic: 53.46, *p*-value: <2.2 × 10^−16^) followed by a Tukey pairwise comparison (differences with *p* < 0.05 have different letters).

The general mass-scaling exponent (ME) calculated for *H. didactylus* was 0.96 +/− 0.08 (Figure 1a) at 15 °C. For each of the 16 fish in the SMR/RMR trial, Figure 1b presents the individual variations of MR. This figure was built using ME. As expected, there was no observable pattern in the weight–MR relationship, meaning that the general ME fit well with the data. Mean MR was 26.44 mg O_2_ kg^−1^h^−1^. Three individuals presented a higher-than-average MR (*p* < 0.05) (Figure 1b) partly due to excessive spontaneous movement during measurement.

### 3.2. Metabolic Rates and Aerobic Scope

**Figure 2:**
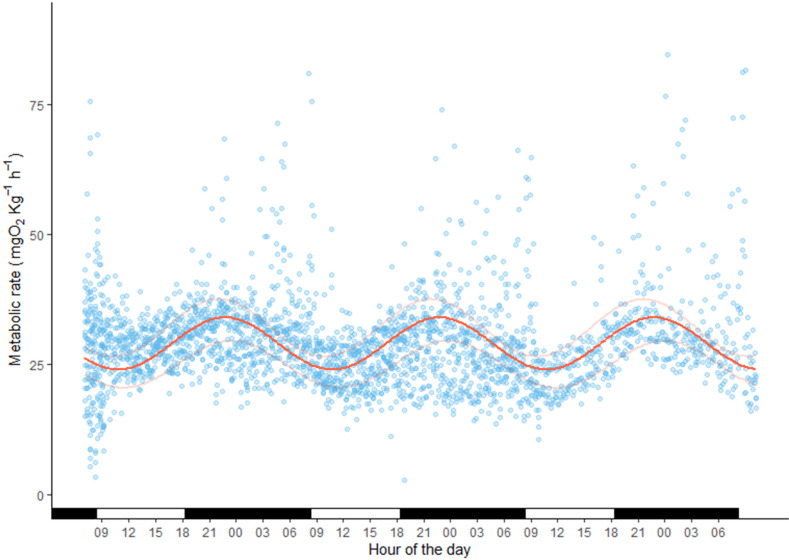
Diel changes in the metabolic rate of *Halobatrachus didactylus* are represented by a scatterplot (each blue point represents a measurement point) with the fitted sine wave model (red line). Dark and light bars indicate the time of light (resting) and dark (active) hours (*n* = 16).

At 15 °C, the SMR of *Halobatrachus didactylus* was estimated at 14.96 mg O_2_ kg^−1^h^−1^ and the RMR at 36.01 mg O_2_ kg^−1^h^−1^. Based on these estimates, the routine aerobic scope for this species was calculated to be 21.05 mg O_2_ kg^−1^h^−1^ (58.5%). In Figure 2, the hourly variations in the MR of the 16 fish that took part in the SMR/RMR determination were plotted for the total duration of 75 h. The daily pattern of higher activity (RMR) and quiescence (mainly SMR) was evident for each 24 h period. The initial highly variable period of approximately 3 h was in accordance with the acclimation-to-respirometer estimation presented in the previous section. This activity pattern of *H. didactylus* can be described by the fitted sine function. Despite the high variability in the data, it was observed that the activity pattern was nocturnal, with peak oxygen consumption occurring at around 21:00, when the fish were actively moving and swimming, followed by a decrease in oxygen consumption as the day progressed, with minimum activity at around 09:00 (Figure 2).

The following function describes the sinusoidal oscillation of the MR of *H. didactylus* throughout the day:

Metabolic rate (mg O_2_ kg^−1^h^−1^)= 5 (+/−0.5) × sine (2 × π × 1 (+/−0.01) × time of day + 5.3 (+/−0.1)) + 29 (+/−3).

**Figure 3:**
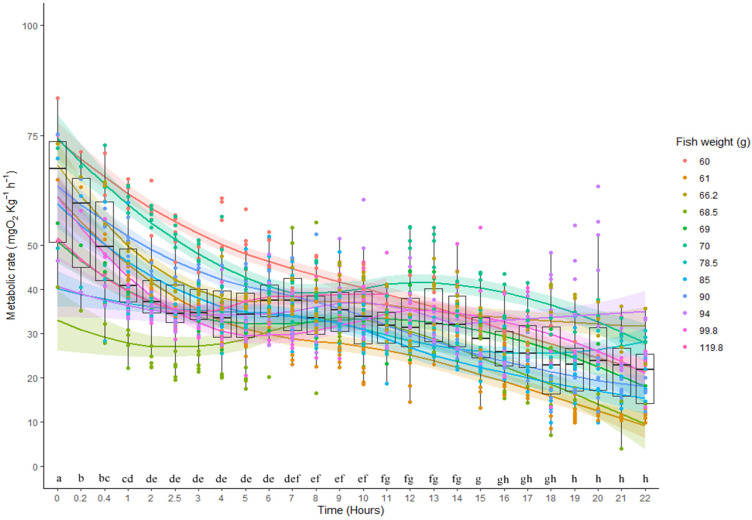
Metabolic rate of *Halobatrachus didactylus* individuals of different weights (lines represent the spline models fitted to the data of each fish) subjected to the exhaustive chase protocol (immediately before hour 0), and subsequent recovery in a 22 h period. Letters show the result of nested Tukey pairwise comparisons (differences with *p* < 0.05 have different letters) between the means of each time range.

Exhaustive exercise produced a marked increase in the MR of *H. didactylus*. We estimated a MMR of 67.31 mg O_2_ kg^−1^h^−1^, producing an aerobic scope of 52.35 mg O_2_ kg^−1^h^−1^ (77.8%). Figure 3 showcases this high MR and the subsequent decrease in a 24 h period. It was evident that after 5 to 6 h, the MR seemed to have stabilized, only to fall to lower values after the 19 h mark. This second decrease in MR might be explained by the dial activity pattern of the species. The SMR estimated using the latter values (20.8 mg O_2_ kg^−1^h^−1^) was close to the SMR estimation produced by the set of individuals used in the SMR trial (Table 1). *H. didactylus* was able to tolerate exhaustive exercise for a median of 22 min before being completely exhausted.

Linear regressions fitted to SMR, RMR, and MMR showed no effect of size on these variables (t = 1.287, *p* = 0.22; t = 0.63, *p* = 0.53; t = −0.723, *p* = 0.48, respectively) as would be expected from employing an appropriate ME. Size and time until exhaustion were not found to have an independent statistical effect on MMR. The full model including the interactions between size and time was not found to be different from the null model (F = 0.4406, *p* = 0.7303).

### 3.3. CT_max_

**Figure 4:**
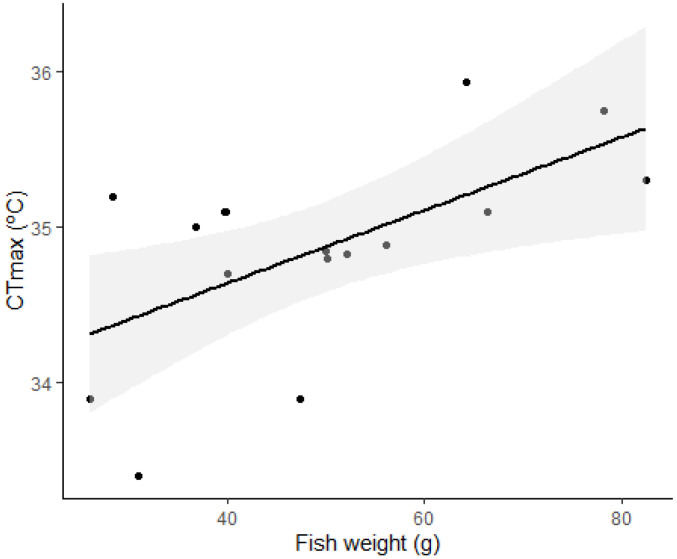
The critical maximum temperature (CT_max_) trial results for *Halobatrachus didactylus*. The line represents the linear relationship between fish weight and CT_max_, and the greyed area the standard error (*n* = 16).

*Halobatrachus didactylus* presented a mean CT_max_ of 34.82 +/− 0.66 °C (*n* = 16). Results from the CT_max_ trial are summarized in Figure 4. The linear relationship of CT_max_ and weight is described by the function:

CT_max_ = 33.39(+/−0.43) °C + 0.02 (+/−0.008) * Weight. (1)

The fit of the CT_max_ to weight showed a low positive correlation (R^2^: 0.380, *n* = 16, F: 3.094, *p* = 0.039).

### 3.4. Temperature Ramp

**Figure 5:**
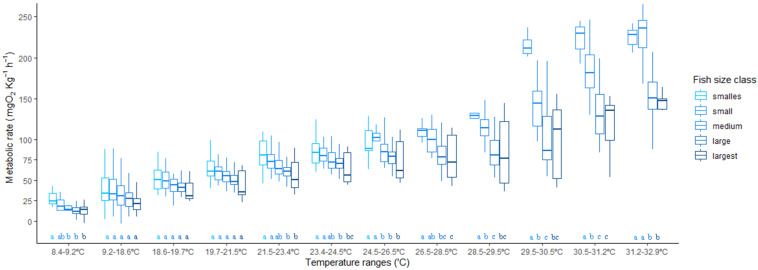
Acute temperature change effect of metabolic rate in different size classes of *Halobatrachus didactylus*. Letters show the results of the nested Tukey pairwise comparisons (differences with *p* < 0.05 have different letters).

The regression tree employed on the temperature ramp data found significantly different MRs at 13 temperature ranges, with a total of 22 tree nodes (Supplementary Material Figure S1a) and at five size ranges, termed as follows: smallest: 13–22.9 g, small: 23–33.9 g, medium: 34–48.9 g, large: 49–140.9 g, largest: 141–159 g (Supplementary Material Figure S1b). More temperature ranges were found in the 9 degrees above 23 °C than in the 11 degrees below, showing a differential effect of temperature on the MR of this species at higher temperatures. In Figure 5, these differences are showcased for each fish size considered. As the temperature increased, the size effect of the fish became more variable and higher at a steady pace. Beyond 29 °C, the MR of *H. didactylus* became increasingly higher and variable. The large and largest animals were consistently similar in their responses to increased temperature, with smaller animals being more variable, and consistently differently affected by temperature increases, as can be observed by the statistical differences in Figure 5. Smaller fish seem to be more susceptible to increased temperature, showing increased metabolic rates and higher variability in their responses. The smallest fish had to be taken out of the chambers before the experiment could be concluded due to the loss of equilibrium at 26 °C.

### 3.5. P_crit_

**Figure 6:**
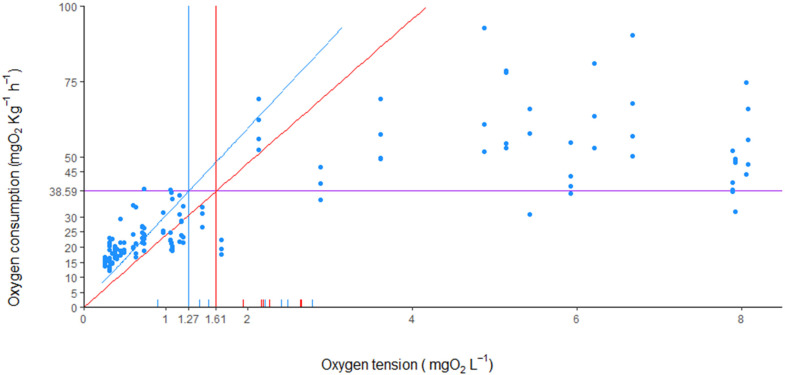
Critical oxygen tension (P_crit_) estimation for eight specimens of *Halobatrachus didactylus* using the LLO (blue lines, P_crit_ = 1.27 mg O_2_ L^−1^) and α methods (red lines, P_crit_ = 1.61 mg O_2_ L^−1^). The purple line indicates the mean standard metabolic rate calculated from all fish used.

Critical oxygen tension for *H. didactylus* was estimated at 1.61 +/− 0.36 mg O_2_ L^−1^ using the α method, and 1.27 +/− 0.68 mg O_2_ L^−1^ using the LLO method, for fish acclimated at 18 °C (Figure 6). Single regression lines (MR versus PO_2_) were plotted through the oxygen-limited portion of the curve and intersected with individual SMR established at high PO_2_. The results obtained using both methods showed P_crit_ to range between 0.59 and 1.97 mg O_2_ L^−1^. The average SMR for these trials was 38.59 mg O_2_ L^−1^ Kg^−1^. Fish weights ranged from 43.7 to 81.0 g.

## 4. Discussion

In this article, we explore the responses of *H. didactylus* to two of the most typical climate change abiotic stressors. We profiled the metabolic performance of this species, providing the first estimates of SMR, RMR, MMR, aerobic scope, CT_max_, P_crit_, and daily aerobic activity. Aside from these physiological parameters, we also provide reference methodological information such as the first ME estimation, acclimation times, and significant temperature ranges for use in future studies concerning this and other closely related species. Toadfishes have been used as models in several metabolism-related studies in the past 50 years (e.g., [59,60,61,62,63]), but there is little information on the active metabolic rates and responses to environmental changes of toadfishes. Indeed, this is, in fact, to our knowledge, the first study to show the whole organism metabolic rates in relation to environmental temperature and oxygen levels, in any member of the Batrachoididae family, since the seminal work of [64], later revisited by Ultsch et al. [18], evaluating the oxyconformity, and by Haschemeyer [65], who studied the impact of temperature in the oyster toadfish *Opsanus tau*. Work on the genus *Opsanus* has been more prolific, and studies dealing with nitrogen excretion and the metabolic costs of sound production have been published ([60,61], respectively). In the present study, the measured SMRs in *H. didactylus* were rather low, 14.96 mg O_2_ kg^−1^h^−1^ in fish maintained at 15 °C, a temperature that reflects the median of their natural thermal amplitude in the wild. The mass specific SMR value of *H. didactylus* is only about half of that measured earlier in the taxonomically related *Opsanus tau* at 21–22 °C [18,61,65], and is surprisingly close to the tropical marine stonefish *Synanceia verrucosa* [66], described as one of the most motionless fish species for which SMR has been estimated. While the reported value of 24 mg O_2_ kg^−1^h^−1^ for *S. verrucosa* is higher, it was measured at 24 °C; assuming a Q_10_ of 1.8 [46]; this value becomes 13.3 mg O_2_ kg^−1^h^−1^ at 15 °C. Other Batrachoidiformes such as *Opsanus tau* (24.5 mg O_2_ kg^−1^h^−1^ at 15 °C; [61]) and *O. beta* (53.3 mg O_2_ kg^−1^h^−1^ at 15 °C; [60]) were found to have higher MR than *H. didactylus*, which in the case of the former was much closer than the latter to the values calculated in this study. Table S1 compiles the SMR values from several papers dealing with intertidal sedentary fish from around the world, from Antarctic to tropical fish. Values were converted from the original units to mg O_2_ kg^−1^h^−1^ at 15 °C (using the suggested Q10 of 1.8 from Norim and Clarke [46] when possible; otherwise the study was not included in the table. More complete resources compiling SMR of several fish species exist (e.g., [39]), Table S1 is only intended to provide the general reference values.

Additionally of note is the relatively fast acclimation shown by this species to a respirometry chamber. General recommendations are around 24 h of acclimation; however, for *H. didactylus*, 3 h was enough (Figure 2), similar to the stonefish [66], making it extremely convenient for use in stress-related studies. This crepuscular species mainly remains still during the daylight hours, even in the respirometry chamber, which allowed us to record such low SMR values. It became more active during lowlight, consuming almost 30% (Table 1) more oxygen at night. Recently, Pereira et al. [35] used acceleration acoustic tags to explore the behavioral patterns of *H. didactylus*, finding that it was much more active during nighttime, a finding that is now supported by our results. Night activity would be compatible with foraging behavior as this species is not solely a sit-and-wait ambush predator, but also actively engages in prey searching [35,67]. The authors of Costa [68] suggest that *H. didactylus* performs daily movements according to temperature, light, and tide. In our study, the temperature was kept constant, and an artificial 11:13 h of day–night lighting (emulating that of the natural environment at Faro, Portugal for January–February) was employed, and the activity pattern inferred from the MR data shown in Figure 2 closely followed this light oscillation, providing evidence that daylight plays a major role in driving the activity patterns in *H. didactylus*. It would be interesting to evaluate whether these circadian oscillations would change in magnitude if the temperature fluctuates according to natural daily variations. It is likely that our estimation of RMR is at the lower end of the activity spectrum for this fish, considering that we only found an increase of about 30% (Table 1) from the basal energy expenditure during the active phase of the day. In natural conditions, where the individuals are able to freely roam and engage in more complex behavior, it is likely that their RMR would be much higher.

Benthic fish species present, in general, lower MMR when compared to species living in the pelagic zone, possibly due to different behaviors related to prey capture and predator avoidance [12,69]. Consistently, the MMR calculated for *H. didactylus* is similar in magnitude to that of other benthic ambush predators such as *Solea solea* and *Esox lucius* (a more complete list of values for many other species is provided in Norim and Clarke [46]). Several factors, both intrinsic and extrinsic, play different roles in determining the MMR of a given species, and even within a species, individual variations in MMR can be considerable, and they can often be measured consistently [70,71,72]. However, our fish showed no sign of significant individual differences in MMR, which might be due to two methodological causes, aside from a potential intraspecific homogeneity in the MMR of this species: the n might not have been large enough to capture differences, or the size range employed might not have been sufficient. Regardless of intraspecific differences, the MMR obtained positions *H. didactylus* on the less active end of the spectrum for bony fish [46], as was previously suggested based on the SMR obtained. Further telemetry studies (similar to the one performed by Pereira et al. [35], but with a different objective) would be helpful at providing insights on how often this species increases its MR by 70% to reach its MMR, which would clarify the ecological importance of this measurement for *H. didactylus*.

Temperature is a major driving variable for the metabolism of ectotherms, and the increase in global temperature due to climate change is a cause of concern for this very reason. Species that are able to tolerate wide ranges of temperatures in their natural distributions are usually “touching the edges” of their metabolic competence to maintain homeostasis [12,16,73,74,75,76]. In the case of *H. didactylus*, the effects of acute increases in the temperature past 29 °C had a marked effect on their MR (Figure 6). This effect was greater in smaller fish than in larger ones, due probably to the size-related threshold effects [39,77,78] that were not covered by the allometric exponent employed. This may also have relevant ecological consequences: temperature is an important reason for the distribution of the species in estuaries and coastal lagoons. A study by Cotter et al. [79] revealed that the conditions responsible for higher densities of small individuals including recruits, in the Tagus Estuary, were mainly high water temperature, low water flow, and the predominance of a muddy substrate. In Ria Formosa, smaller fish are more abundant in shallow, warm water areas of mud or seagrass beds, but are unlikely to be exposed to extreme elevated temperatures. Meanwhile, larger juveniles often find shelter in shell concretions, abandoned bricks, or other debris, while larger males, which like other batrachoidids build nests and guard the eggs [35], may be exposed to high temperatures in very shallow water during low tides. Indeed, the calculated CT_max_ values (34.61–35.39 °C) were not much higher than the extreme temperatures these fish may encounter in such conditions, but the virtual absence of mortalities after the experimental procedure suggests that at the moment, the species is able to cope well with the acute changes in temperature present in their environment. This situation may change in the near future, with the increase in water temperature levels [4], which makes a mechanistic understanding of the thermal responses of fish essential to predict the vulnerability of species and populations to climate change [80].

Describing the oxygen dependence in marine fish is relevant in the context of climate change, where the geographic extension and intensity of aquatic hypoxia has increased [20,81,82,83]. This phenomenon is even more relevant in shallow coastal lagoons, where elevated temperatures may contribute to low DO. Recently, there has been some discussion as to which methodological and statistical approach to employ in estimating P_crit_, and details about several possible methodologies are available and have been discussed extensively [20,24,83]. *H. didactylus* proved to be resilient to hypoxia, as evidenced by the low P_crit_ estimated. The bibliographic search revealed only two papers describing hypoxia tolerance measured as oxygen consumption for other batrachoid fish: Ultsch et al. [18] presented similar low P_crit_ results for *Opsanus tau*, 1.43 mg O_2_ L^−1^, and Craig et al. [84] provided no estimate, but rather categorized *Porichthys notatus* as a complete oxyconformer. LeMonie et al. [85], also working with *P. notatus*, described a high hypoxia tolerance when fish were subjected to 8% oxygen saturation at 11 °C; however, no estimate of P_crit_ was supplied. In our trials, all individuals of *H. didactylus* fully recovered after the trials that exposed them to an oxygen concentration lower than 1 mg O_2_ L^−1^. Other species with similar P_crit_ have shown substantially delayed mortality after similar procedures [86,87,88,89], which further showcases the resilience of *H. didactylus* to hypoxia. In fact, the species is known to survive for long periods exposed to air, either in shallow tide pools, sandbanks, or in market benches, which also raises questions about the ability of the species to perform aerial or cutaneous respiration. Studies have been done onthe taxonomically close *P. notatus* [90,91] and the findings indicate low, but ecological relevant, aerial respiration in males, collected while guarding egg nests exposed during extremely low tides [92]. In said study, *P. notatus* consumed O_2_ and released CO_2_ continuously into the air throughout emergence, but the fish remained quite inactive and showed the lowest mass-specific metabolic rates of all species tested [90]. The mechanisms for aerial respiration were not explored, and the possibility of cutaneous O_2_ exchange is unknown for *H. didactylus*, neither during air exposure nor when immersed. In the current case, as the decrease in O_2_ concentration was acute, it is unlikely that any structural reconfiguration of gill surface [93] or increase in hemoglobin–O_2_ binding [94] takes place, as shown in hypoxia-acclimated experiments on other species [95,96]. Instead, we believe that metabolic depression (e.g., [97]) would be the likely mechanism underlying *H. didactylus*’ resilience [98,99,100,101]. This phenomenon has been shown to occur in other related species of Osteichthyes [102,103,104] and Chondrichthyes [105,106,107], and may also help *H. didactylus* to survive when exposed to air. While further research employing biomarkers and blood physiology studies are needed to deepen our understanding of hypoxia tolerance in this species, P_crit_ has scientific importance, as it allows for predictive statements [108].

The increase in water temperatures and oxygen minimum zones has caused changes in the distribution of many fish species [109,110] and may also induce a shift in this species distribution. *H. didactylus* has a complex reproduction process, with egg nests, parental care, and lack of a pelagic larval phase, and a seemingly sedentary, site-dependent life-style [111], which may suggest changes in its distribution to be unlikely. However, studies using acoustic transmitters have shown that particular individuals can actually cover quite some ground [112], and eventually colonize new areas along the coastline. Until recently, the Tagus estuary was considered the northern limit of the species distribution, with genetic data pointing to a very recent Holocenic (hundreds to thousand years) (re)colonization from the south (e.g., Algarve region, where the present study was conducted), with possible successive colonizations/extinctions driven by changes in temperature [113]. However, such a rate of dissemination could now be increased, as recent surveys have shown that the species can migrate north along the Portuguese and Spanish coast, and there is anecdotal evidence that *H. didactylus* is now captured more often at the Atlantic coast in the north of Portugal and Galicia [114]. Their remarkable tolerance to high temperatures and hypoxia found in the present study, certainly attest to their adaptability to new environments. Cases of isolated individuals captured as far as Greece have already been reported [115].

It has been suggested that a physiological trade-off between a fast and slow lifestyle exists in fishes [12,38]. Sedentary species would present low oxygen demands and be more resistant to thermal stress and hypoxia (as described for the plain midshipman [92]). From the metrics presented in this article, we can conclude that *H. didactylus* is highly resilient to acute environmental variations in temperature and oxygen content, which might translate to a good acclimation capacity when exposed to chronic, rather than acute, stressors. Studies employing an experimental setup that replicates chronic exposure to high temperature and hypoxia would be very useful to understand the acclimation potential of this and other resident species (good examples of such setups can be found in Di Santo [116] and McArley et al. [117]). Furthermore, studies combining respirometry with biomarkers such as oxidative stress enzymes, damaged DNA, and hematological parameters [45,118] would also provide a more physiologically complete panorama of how *H. didactylus* and other batrachoids cope with the unpredictable and variable world they thrive in.

## 5. Conclusions

*Halobatrachus didactylus* is an extremely sedentary fish, with one of the lowest standard metabolic rates found in temperate fish (SMR: 14.96 mg O_2_ kg^−1^h^−1^). *H. didactylus* activity increases at night, when its metabolic rate increases drastically (RMR: 36.01 mg O_2_ kg^−1^h^−1^). The maximum metabolic rate of this species was estimated to be 67.31 mg O_2_ kg^−1^h^−1^, producing an aerobic scope of 52.35 mg O_2_ kg^−1^h^−1^ (77.8% increase). This batrachoid is highly resistant to thermal and hypoxia stress, with a CT_max_ of 34.82 +/− 0.66 °C and P_crit_ ranging from 0.59 to 1.97 mg O_2_ L^−1^. We found size-specific differences in this stress response, with smaller individuals being more sensitive. The metrics obtained in this study prove that *H. didactylus* is remarkably resilient to acute environmental variations in temperature and oxygen content, which might enable it to adapt to the extreme abiotic conditions forecasted for the world’s oceans in the near future. Future studies looking into the chronic effects of increased temperature and hypoxia would help complete our understanding on the remarkable resilience of this species. 

## Figures and Tables

**Table 1:** Standard, routine, and maximum metabolic rate (SMR, RMR, and, MMR, respectively in mg O_2_ kg^−1^h^−1^) of *Halobatrachus didactylus* subjected to an exhaustive chase protocol as well as aerobic scope and routine aerobic scope (%M/S and %R/S), calculated as a percentage. Weight (in g) and time being chased until exhaustion (in minutes). Median values are provided in the last row.

Fish No.	Weight	MMR	RMR	SMR	%M/S	%R/S	Time
1	60	83.4	29.7	22.6	73.0	24.0	18.0
2	61	64.9	17.8	10.6	83.7	40.6	23.0
3	66.2	73.1	35.8	28.5	61.0	20.4	15.0
4	68.5	40.6	24.1	12.4	69.4	48.6	30.0
5	69	55.1	28.1	19.1	65.3	32.0	15.0
6	70	72.1	37.0	29.1	59.6	21.3	25.0
7	78.5	49.4	27.1	22.9	53.7	15.4	26.0
8	85	69.8	22.0	13.5	80.7	38.5	19.0
9	90	75.3	24.5	16.0	78.8	34.6	28.0
10	94	46.6	33.5	27.2	41.7	18.8	16.0
11	99.8	75.1	29.6	23.4	68.9	21.2	24.0
12	119.8	51.1	38.9	18.3	64.2	53.0	21.0
Median	74.3	67.3	28.9	20.8	69.1	27.8	22.0

## Data Availability

Data used in this article are available on the VLIZ website and can be found here: “Molina, J.M.; Guerreiro, P.M.; Argentine Institute of Oceanography (IADO): Argentina; Centre of Marine Sciences (CCMAR): Portugal; (2021): Measurements of respiratory metabolism in *Halobatrachus didactylus*. Marine Data Archive. https://doi.org/10.14284/503“.

## Supplementary Materials

The following supporting information can be downloaded at: https://www.mdpi.com/article/10.3390/ani13040632/s1, Figure S1: Regression tree analysis applied to a) temperature ramp and b) weight data of Halobatrachus didactylus. Separation nodes (bubbles) present the p of the statistical test, between the ranges (numbers on the connections). End nodes present total data points and their distribution on an individual boxplot.; Table S1: Standard metabolic rates (SMR) of several sedentary fish. SMR at 15 °C was calculated using the suggested Q10 of 1.8 [46]). Refs. [119,120,121] are cited in Supplementary.
