# Supplementary material for: Metabolic Responses and Resilience to Environmental Challenges in the Sedentary Batrachoid Halobatrachus didactylus (Bloch & Schneider, 1801)
Source: Animals (Basel). 2023 Feb 11;13(4):632. doi: 10.3390/ani13040632 (PMC9951689; doi:10.3390/ani13040632)
Supplement: Supplementary file 1 [file animals-13-00632-s001.zip › animals-2153971-supplementary.pdf]

Supplementary Materials:

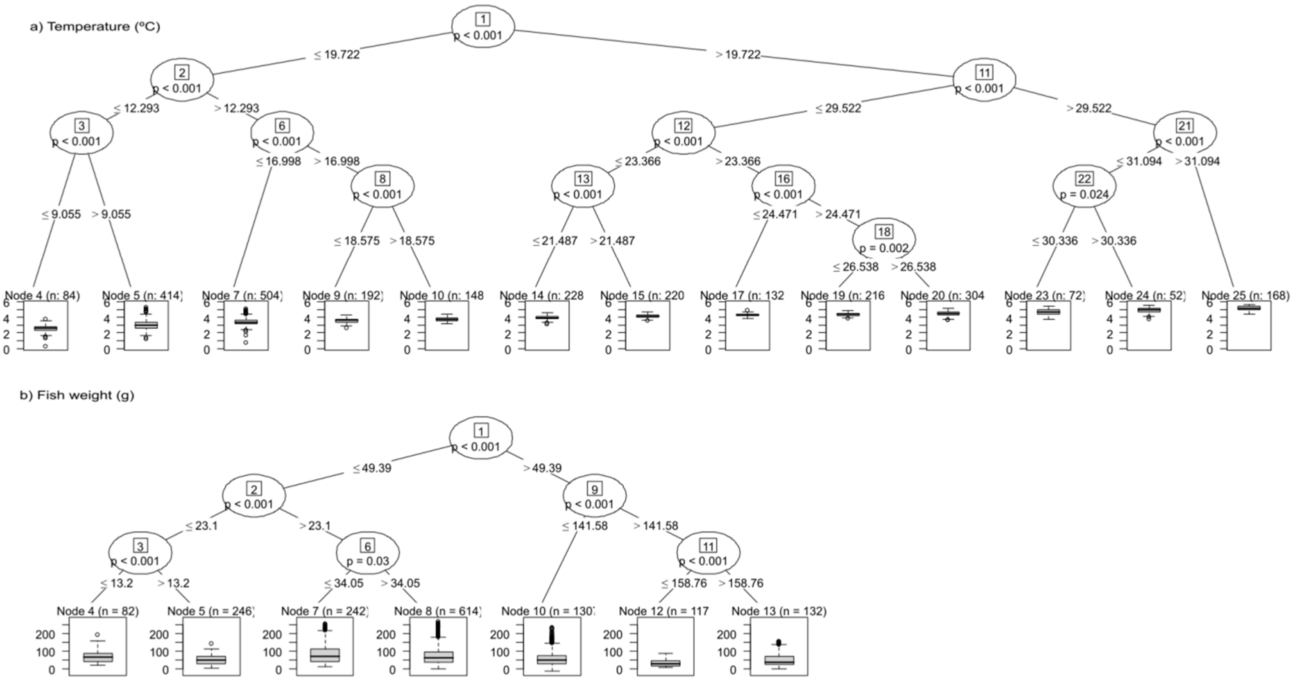

Figure S1. Regression tree analysis applied to a) temperature ramp and b) weight data of *Halobatrachus didactylus*. Separation nodes (bubbles) present the p of the statistical test, between the ranges (numbers on the connections). End nodes present total data points and their distribution on an individual boxplot.

Table S1. Standard metabolic rates (SMR) of several sedentary fish. SMR at 15 ºC was calculated using the suggested Q10 of 1.8 [46]).

Species	SMR (mgO2 Kg-1 L-1)	Weight (g)	Temperature (ºC)	SMR at 15 ºC (mgO2 Kg-1 L-1)	Order	Study
Opsanus tau	27.48	214-612	22	18.2	Batrachoidiformes	Ultsch et al., 1981 [18]
Opsanus tau	35	310-437	21	24.5	Batrachoidiformes	Amorim et al 2001 [61]
Opsanus beta	96	50-230	25	53.3	Batrachoidiformes	Gilmour et al 1998 [60]
Notothenia neglecta	26	22-63	0	62.8	Perciformes	Johnston et al., 1991 [119]
Agonus cataphractus	42	43-313	4	80.2	Scorpaeniformes	Johnston et al., 1991 [119]
Oligocottus maculosus	39.4	1.56-1.78	12.2	46.4	Scorpaeniformes	Sloman et al 2008 [120]
Bellapiscis medius	160	6.05-6.33	15	160	Blenniformes	Hilton et al., 2008 [118]
Bellapiscis lesleyae	180	2.99-4.21	15	180	Blenniformes	Hilton et al., 2008 [118]
Myoxocephalus scorpius	48	27-164	7	76.8	Scorpaeniformes	Johnston et al., 1991 [119]
Paracirrhites arcatus	68	13-36	24	40.1	Perciformes	Johnston et al., 1991 [119]
Synanceia verrucosa	24	177	25	13.3	Scorpaeniformes	Kunzmann 2021 [66]
Scorpaenidae gen. sp	32.3	18-82	25	17.9	Scorpaeniformes	Zimmerman and Kunzman 2001 [44]
Tautogolabrus adspersus	20.2	87.3-100.1	6.2	33.9	Labriformes	Speers−Roesch et al., 2018 [121]
Halobatrachus didactylus	16.3	14-148	15	16.3	Batrachoidiformes	This study
